# From means to meaningful: undertaking cluster analysis to develop health literacy profiles of people in Australian prisons

**DOI:** 10.1371/journal.pone.0351554

**Published:** 2026-06-11

**Authors:** Scott W. Gill, Christina Cheng, Julia Bowman, Caron Shaw, Richard H. Osborne

**Affiliations:** 1 Global Health and Equity Development Hub, Violet Vines Marshman Centre for Rural Health Research, La Trobe Rural Health School, La Trobe University, Melbourne, Australia; 2 Justice Health and Forensic Mental Health Network, Sydney, Australia; 3 Patient Safety Team, Clinical Excellence Commission, Sydney, Australia; 4 Department of Public Health, University of Copenhagen, Kobenhavn, Denmark; Tarbiat Modares University Faculty of Medical Sciences, IRAN, ISLAMIC REPUBLIC OF

## Abstract

Prison populations are diverse, experience substantial vulnerabilities and commonly have poor health outcomes. Cluster analysis of Health Literacy Questionnaire (HLQ) data provides an opportunity to account for the prison population’s diversity and generate fit-for-purpose intervention ideas for the prison context. This mixed-methods study aimed to undertake cluster analysis to explore the diverse health literacy profiles of people in the New South Wales (NSW), Australia, prison setting, and to co-design a series of vignettes that represent people in prison. Cluster analysis using Ward’s method was applied to HLQ data (n = 471) to identify health literacy profiles of people in NSW prisons. Semi-structured interviews with people in prison were conducted to collect in-depth personal experiences of prison health services access and use. Interview data were combined with cluster analysis results to co-design vignettes with various stakeholders. A 14-cluster solution was identified as the optimal solution for the sample as a whole, whereas a 9-cluster solution was identified for the sample of people who self-identified as Aboriginal. Qualitative data from 10 semi-structured interviews were combined with the health literacy profiles identified in the cluster analyses. A total of 23 vignettes were co-designed with stakeholders (n = 34) to represent the voices of a typical person with lived experience across each cluster. Cluster analysis has unmasked previously unforeseen variations and enabled us to move from merely reporting means to gaining meaningful insights into the health literacy profiles of people in prisons. Our findings are important because they show that using the mean or proxies alone does not capture population variation, which may inadvertently exclude or disadvantage population subgroups. The co-designed vignettes are expected to stimulate discussions to generate localised, fit-for-purpose health literacy-informed interventions to address health inequities for prison populations in NSW.

## Background

People in prison commonly experience a substantial burden of disease and poorer health compared with general populations [1–3]. People in New South Wales (NSW) prisons, Australia’s most populous state, have higher rates of mental health conditions [4–8], Hepatitis B, C and HIV [4–6,9], drug and alcohol disorders [5,6] and other physical health conditions [4,5,7,8] compared with the general population. This health burden is compounded further by high rates of recidivism, with nearly one-third (31%) of people released from custody reincarcerated within 2 years [10].

The NSW prison population is not a homogeneous group. Aboriginal and Torres Strait Islander people (hereafter respectfully referred to as Aboriginal people) make up approximately one-third of the prison population [11], representing a heterogeneous collection of hundreds of groups, each with their own language, history, and cultural norms [12]. Moreover, it has been reported that one in five people in NSW prisons are from Culturally and Linguistically Diverse (CALD) backgrounds, with over 118 different cultural backgrounds represented [13]. Additionally, people vary in age, sex, legal status, educational attainment and offence type [14]. This observed heterogeneity poses challenges for intervention design, as approaches that fail to account for this diversity risk overlooking population subgroups with distinct strengths and challenges.

### Health literacy and its potential to inform intervention development

Health literacy is a complex, multi-dimensional concept [15,16] that is an important factor in enabling individuals and communities to manage their health successfully [17] and has been described as a modifiable social determinant of health [18]. Health literacy refers to an individuals’ knowledge, confidence, and comfort with accessing, understanding, appraising, remembering, and using health information [17]. Health literacy development builds on the concept of health literacy to create enabling environments to support people to “access, understand, appraise, remember and use health and healthcare” [17 p. 2, Box 2]. To achieve health literacy development in a population, it is first necessary to understand the breadth of health literacy strengths and challenges of individuals across the population of interest. This can be achieved by applying specifically designed health literacy needs assessments that capture diverse independent health literacy indicators across the population of interest.

Despite many questionnaires existing to measure one or more domains of health literacy, there is only one questionnaire, the Health Literacy Questionnaire (HLQ) [15], which was developed to understand patterns of health literacy strengths and challenges in populations [19]. The HLQ is a 44-item health literacy measurement tool that measures the concept through nine independent but complementary domains of health literacy [15]. The HLQ is commonly used as a needs assessment to understand potentially modifiable health literacy mechanisms that may exist across individuals and communities.

In our 2023 study undertaken in 14 metropolitan NSW prisons, we found that the average HLQ scale scores among 471 people in prison were substantially lower than those of the general Australian population [20]. We found that all 9 HLQ scales were sufficiently reliable (Cronbach’s alpha ranged from 0.7 to 0.9) [20]. Differences were also observed according to sex and legal status, with females and those on remand (i.e., those not sentenced) having more health literacy challenges compared with their counterparts [20]. While these findings highlight overall challenges, analyses based on mean scale scores or general participant characteristics (e.g., sex, age, education) do not reveal whether specific subgroups exist that may have particular strengths or challenges [21], which is critical for a contemporary strengths-based approach to clinical practice, service redesign or community development. As pointed out in recent WHO recommendations [22], taking a one-size-fits-all approach may be effective for community members with average levels of a trait, but such approaches may inadvertently marginalise those who are not average, and interventions based on population averages may ultimately increase inequalities.

Cluster analysis has been proposed as a robust method to reveal and make explicit the health literacy strengths and challenges of diverse groups of people in the population of interest [21], and is a core method employed in the Ophelia (Optimising Health Literacy and Access) process [23–25]. To date, health literacy development methods, including cluster analysis as part of the Ophelia process, have been applied in many diverse settings, including among populations that experience marginalisation [26–32]. However, to our knowledge, cluster analysis of health literacy data from people in prison has not previously been undertaken.

The current paper details one component of a large study being undertaken in the Australian prison context using the Ophelia process [33]. Specifically, this study aimed to conduct cluster analyses of HLQ data to identify if distinct health literacy profiles exist among people in NSW prisons, and if so, co-design vignettes (evidence-based stories) that represent the breadth of the people in prison to support intervention development.

## Methods

**Fig 1:**
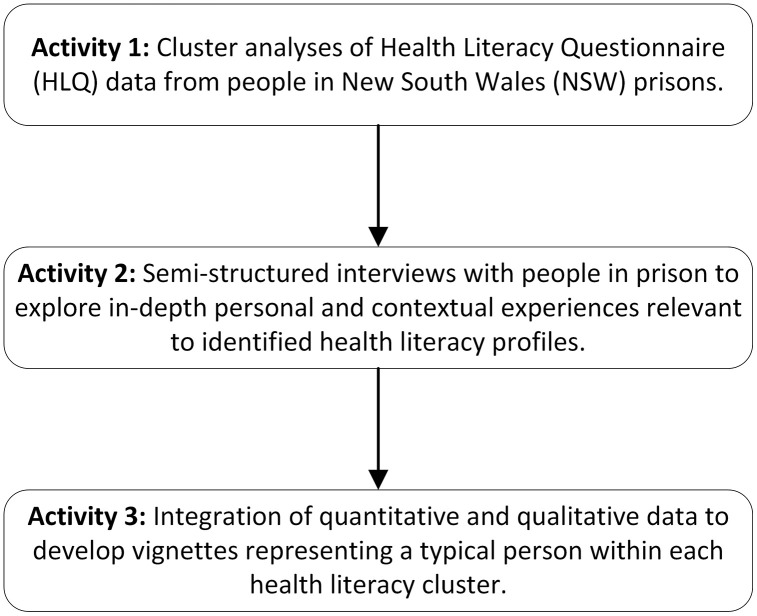
Overview of study activities and methods.

This study employed a mixed-methods design comprising three research activities (Fig 1). Activity 1 involved cluster analysis of previously collected HLQ survey data from 471 people in NSW prisons. Semi-structured interviews were undertaken to collect in-depth personal experiences and insights into why people responded to HLQ items the way they did (Activity 2), which were then combined with the cluster analysis health literacy profiles to inform vignette creation (Activity 3). Each activity, where possible, included co-design and extensive stakeholder engagement.

### Activity 1: Cluster analyses of HLQ data

#### Data source.

The recruitment and survey methods have been described in detail elsewhere [20,34]. Briefly, 471 people across 14 metropolitan correctional centres who had accessed a primary care nurse at the correctional centre were recruited. Participant lists were stratified by correctional centre health clinic and Aboriginal identity. People in prison were eligible to participate if they had accessed the Justice Health NSW primary care nursing service between 1 October 2019 and 30 September 2020, could comprehend the study procedures in English and were able to provide written informed consent [20,34]. Following consent, the survey, including demographic questions and the HLQ, was administered verbally.

The HLQ comprises nine scales, each with four to six items. The first five scales use four response options from strongly disagree (1; lowest) to strongly agree (4; highest). The last four scales use five response options from cannot do or always difficult (1; lowest) to always easy (5; highest). Scale scores were calculated by summing the scores of items in the scale and dividing by the number of items. The HLQ does not generate an overall mean score, with each mean scale score reported separately. The nine scales of the HLQ are [15]:

1. Feeling understood and supported by health care providers (four items)
2. Having sufficient information to manage my health (four items)
3. Actively managing my health (five items)
4. Social support for health (five items)
5. Appraisal of health information (five items)
6. Ability to actively engage with health care providers (five items)
7. Navigating the health care system (six items)
8. Ability to find good health information (five items)
9. Understand health information enough to know what to do (five items)

#### Data analysis.

The statistical software program IBM SPSS Statistics Version 29 [35] was used to run hierarchical cluster analysis using Ward’s method [36] as recommended in the Ophelia process [21,23–25]. Cluster analysis is a multivariate technique that groups individuals on a particular set of characteristics (i.e., the nine HLQ scales in this study), with Ward’s method using the sum of squares between clusters summed over all variables [37]. Two separate analyses were undertaken: 1) the overall participant sample (n = 471) and 2) participants who self-identified as Aboriginal (n = 97). The second analyses were undertaken due to the overrepresentation of Aboriginal people in custody and to ensure we acknowledge that Aboriginal people may have different cultural views and needs for their health. For each cluster, HLQ scale scores were presented as means and standard deviations (SDs) and demographic characteristic data as percentages.

Previous papers, most notably Cheng et al. [21] have recommended procedures to determine the most appropriate number of clusters in a cluster solution. Solutions were guided by patterns in HLQ scale scores, supported by the SDs and related participant demographic characteristics [23]. A SD of 0.6 for any HLQ scale was assumed to indicate substantial variation for that scale within a cluster. However, clusters with small participant numbers will likely have greater variation (i.e., a higher SD) across scale scores. We also examined participants’ demographic characteristics data and health profiles to ensure that potential clusters with similar health literacy patterns, but different demographic characteristics and health profiles were represented in the analyses, enabling different intervention strategies to be considered to support the diverse groups represented. A strong determinant of the number of clusters including an analysis of the pattern exhibited in the two clusters that arise from a parent cluster split in each successive step. That is, when, for example, the data split from 9 to 10 clusters. Each new consecutive pair arising from the parent cluster were examined to identify if any differences between the health literacy patterns are also linked to unique demographic and clinical patterns. This examination and analysis continued until further splits no longer provided meaningful patterns in the data.

### Activity 2: Semi-structured interviews

#### Participant recruitment.

Semi-structured interviews were conducted with people in prison who had previously been involved in broader research activities and had completed at least one part of the HLQ. Potential participants were invited to participate using a purposive sample approach [38], based on the cluster analyses results, to capture varying health literacy profiles. Individual HLQ scale scores from previous participants were compared with cluster profiles, and those with similar scale score patterns were invited to participate in an interview. Given that the purpose of the interviews was to explore experiences and understand why people responded to HLQ items the way they did, recruitment to data saturation and sample size calculation were not undertaken. Given our previous experience 10 people were invited to participate as this number was anticipated to provide a rich range of experiences to assist with drafting realistic narratives around the cluster profiles.

#### Data collection.

Semi-structured face-to-face interviews were undertaken across five prisons in November and December 2023. Each interview took about 30 minutes; the interviews explored health literacy strengths and challenges to gain insights into the population’s experience of accessing and using health services in prison (S1 File). Questions were also asked specifically about barriers to health care, connecting to healthcare providers, privacy, and whether they had support to access healthcare in prison. Based on the participants’ HLQ scores, further questions were asked about why they scored low on some scales. Interviews were audio-recorded and transcribed.

#### Data analysis.

Semi-structured interview data did not undergo thematic analysis but were mainly used to supplement the cluster analysis results for vignette development.

### Activity 3: Vignette development

The identified health literacy profiles and associated demographic characteristic data were combined with qualitative data from the semi-structured interviews to develop vignettes representing a typical person for each cluster.

Vignettes were initially developed by synthesising quantitative health literacy and demographic data within each individual cluster. For each cluster, all nine HLQ scale scores and associated demographic characteristics were collated and organised under four headings: demographics, health status, education, and health literacy. These formed the basis of the initial vignette drafts, with HLQ scale descriptions and item content used to translate the quantitative scores into narrative descriptions of health literacy strengths and challenges. Each vignette was assigned a first name chosen to be a common male or female for a person of a similar age.

Qualitative data from the semi-structured interviews were then used to improve the contextual relevance of the vignettes. Interview transcripts were reviewed to identify commonly used phrases, vernacular, and descriptions of routine health-care experiences. For example, references to “pill parade”, “going to the clinic”, “walking in the yard” or being given Panadol by nurses. Particular attention was paid to how participants described their reasoning for, and experiences of, specific health literacy challenges or strengths. These qualitative elements were incorporated to reflect how people in prison describe and experience health care services.

Vignettes were designed to depict the wide range and diversity of health literacy strengths and challenges across the participants and subsequent profiles. Each vignette was designed to convey the persona’s strengths and challenges as an evidence-informed story about these different experiences and potential mechanisms that influence people in NSW prisons to access, understand and use health information and services. Vignettes were initially written in the third person, following Ophelia process recommendations [23–25]. In keeping with co-design principles, an iterative engagement process with staff and people in prison was conducted throughout their development. Between February and April 2024 draft vignettes were presented to these stakeholder groups in up to four rounds of engagement (depending on the group) and were progressively updated to incorporate their suggestions for improvement.

### Ethics

This study was undertaken in accordance with the National Statement on Ethical Conduct in Human Research 2007 (updated 2018), a statement of ethical principles for research involving human participants in Australia, which is underpinned by the Declaration of Helsinki. Ethics approval was obtained from four Human Research Ethics Committees that preside over the study setting and the researcher’s institutions. These included the Justice Health and Forensic Mental Health Network Human Research Ethics Committee (HREC) (Reference: 2022/ETH01433), the Aboriginal Health and Medical Research Council HREC (Reference: 2007/22), the La Trobe University HREC (Reference: 2022/ETH01433), and the Corrective Services Ethics Committee (Reference: D2022/145326). In accordance with Chapter 2.3 of the National Statement on Ethical Conduct in Human Research, a waiver of consent has been granted to access and undertake secondary analysis of the previously collected HLQ data. All participants involved in semi-structured interviews provided written informed consent.

## Results

### Activity 1: Cluster analyses of HLQ data – cluster solution for the overall prison sample

A 14-cluster solution was selected as the optimal cluster solution for the overall sample. Table 1 describes the HLQ scale scores and associated participant demographic data for the overall sample mean and cluster solutions. Each cluster is described below. A title is given to each cluster that reflects the distinguishing features of the cluster and demographic pattern.

**Table 1:** Overall sample mean and health literacy profiles based on the 14-cluster solution.

Clusters		Mean	#1	#2	#3	#4	#5	#6	#7	#8	#9	#10	#11	#12	#13	#14
Number of participants in the cluster n (%)		**471** **(100)**	23 (4.9)	20 (4.2)	27 (5.7)	27 (5.7)	77 (16.3)	82 (17.4)	27 (5.7)	36 (7.6)	44 (9.3)	32 (6.8)	14 (3.0)	32 (6.8)	24 (5.1)	6 (1.3)
**Within cluster HLQ scale mean score (SD)**																
1. Feeling understood and supported by health care providers	Score range: 1–4	**2.70 (0.58)**	3.51 (0.39)	3.01 (0.27)	3.18 (0.47)	3.17 (0.30)	2.87 (0.30)	2.70 (0.36)	3.07 (0.23)	2.97 (0.39)	2.35 (0.39)	2.34 (0.38)	2.32 (0.30)	2.16 (0.29)	1.41 (0.38)	2.08 (0.56)
2. Having sufficient information to manage my health		**2.71 (0.52)**	3.54 (0.33)	3.10 (0.17)	3.13 (0.36)	3.03 (0.36)	2.98 (0.21)	2.66 (0.31)	2.61 (0.40)	2.83 (0.42)	2.61 (0.45)	2.19 (0.35)	2.48 (0.32)	2.16 (0.32)	1.89 (0.32)	1.92 (0.34)
3. Actively managing my health		**3.03 (0.46)**	3.79 (0.24)	3.06 (0.17)	3.68 (0.33)	2.42 (0.33)	3.09 (0.20)	2.89 (0.31)	3.26 (0.40)	2.91 (0.32)	3.09 (0.34)	2.91 (0.23)	2.24 (0.21)	3.01 (0.30)	3.24 (0.42)	1.77 (0.41)
4. Social support for health		**2.59 (0.51)**	3.02 (0.57)	2.96 (0.34)	3.13 (0.40)	2.96 (0.30)	2.85 (0.32)	2.52 (0.35)	2.40 (0.31)	2.80 (0.40)	2.39 (0.44)	2.21 (0.21)	2.47 (0.29)	2.29 (0.36)	1.72 (0.43)	1.50 (0.35)
5. Appraisal of health information		**2.58 (0.49)**	3.37 (0.26)	2.95 (0.22)	2.97 (0.43)	2.67(0.39)	2.92 (0.39)	2.48 (0.32)	2.22 (0.27)	2.66 (0.42)	2.60 (0.27)	2.03 (0.19)	2.29 (0.30)	2.26 (0.30)	2.05 (0.71)	1.57 (0.32)
6. Ability to actively engage with healthcare providers	Score range: 1–5	**3.34 (0.91)**	4.46 (0.47)	4.74 (0.25)	3.74 (0.57)	4.08 (0.46)	3.84 (0.35)	3.70 (0.41)	3.39 (0.50)	3.03 (0.66)	2.54 (0.49)	3.08 (0.42)	2.50 (0.46)	2.05 (0.55)	1.75 (0.47)	1.97 (0.65)
7. Navigating the healthcare system		**3.07 (0.88)**	3.99 (0.63)	4.58 (0.30)	3.33 (0.62)	3.77 (0.27)	3.72 (0.39)	3.35 (0.49)	2.67 (0.41)	2.78 (0.41)	2.38 (0.56)	2.58 (0.60)	2.61 (0.36)	1.84 (0.42)	1.92 (0.52)	1.67 (0.57)
8. Ability to find good health information		**3.09 (0.85)**	4.07 (0.50)	4.49 (0.33)	3.19 (0.45)	3.75 (0.41)	3.76 (0.41)	3.40 (0.48)	2.60 (0.30)	2.73 (0.65)	2.69 (0.46)	2.36 (0.52)	2.49 (0.44)	1.92 (0.39)	2.04 (0.58)	1.70 (0.41)
9. Understand health information well enough to know what to do		**4.01 (0.69)**	4.77 (0.26)	4.83 (0.23)	4.13 (0.56)	4.36 (0.46)	4.12 (0.38)	4.20 (0.40)	4.28 (0.45)	3.05 (0.71)	3.78 (0.59)	4.23 (0.47)	3.17 (0.66)	3.40 (0.55)	4.03 (0.52)	2.10 (0.55)
**Within cluster sociodemographic characteristics**																
Mean age (years)		**41.6**	47.9	42.7	42.3	42.2	41.1	43.4	40.3	39.9	41.5	44.7	37.2	35.9	38.9	42.0
Age categories																
18 to 29 years (%)		**21.9**	13.0	15.0	18.5	25.9	22.1	23.2	22.2	27.8	25.0	12.5	28.6	31.3	16.7	0.0
30 to 45 years (%)		**42.3**	39.1	40.0	51.9	37.0	40.3	39.0	40.7	47.2	36.4	37.5	42.9	50.0	54.2	66.7
45 to 59 years (%)		**25.3**	26.1	40.0	11.1	29.6	27.3	19.5	29.6	19.4	27.3	40.6	28.6	18.8	20.8	33.3
Older than 60 years (%)		**10.6**	21.7	5.0	18.5	7.4	10.4	18.3	7.4	5.6	11.4	9.4	0.0	0.0	8.3	0.0
Females (%)		**18.7**	17.4	15.0	14.8	11.1	15.6	11.0	25.9	30.6	25.0	9.4	21.4	28.1	29.2	33.3
Aboriginal identity (%)		**20.6**	13.0	45.0	14.8	11.1	19.5	17.1	22.2	27.8	18.2	21.9	35.7	18.8	20.8	33.3
English is primary language spoken at home (%)		**86.2**	82.6	90.0	77.8	96.3	85.7	85.4	88.9	83.3	84.1	93.8	85.7	78.1	91.7	100.0
Self-reported a health condition (%)		**52.4**	47.8	50.0	70.4	66.7	40.3	51.2	66.7	38.9	59.1	53.1	50.0	56.3	50.0	66.7
Completed year 10 or below at high school (%)		**59.4**	52.2	70.0	48.1	63.0	70.1	58.5	44.4	72.2	47.7	62.5	100.0	46.9	70.8	66.7
Completed a Bachelor’s degree or above (%)		**11.3**	4.3	0.0	14.8	11.1	7.8	14.6	14.8	2.8	18.1	15.6	0.0	15.6	16.7	0.0
Mean time in custody (years)		**2.8**	4.4	3.3	2.3	3.3	2.7	3.0	3.2	2.6	2.9	2.0	3.6	1.3	4.3	1.5
Serving a custodial sentence (%)		**68.2**	78.3	55.0	63.0	85.2	67.5	67.1	77.8	77.8	68.2	65.6	57.1	62.5	62.5	33.3
Security classification																
Minimum (%)		**43.5**	34.8	40.0	33.3	63.0	49.4	50.0	55.6	38.9	36.4	43.8	28.6	40.6	29.2	16.7
Medium (%)		**18.0**	17.4	15.0	29.6	3.7	15.6	13.4	11.1	33.3	22.7	15.6	28.6	18.8	16.7	33.3
Maximum (%)		**38.4**	47.8	45.0	37.0	33.3	35.1	36.6	33.3	27.8	40.9	40.6	42.9	40.6	54.2	50.0

Notes: The colour coding of cells within clusters uses green to indicate the highest levels (e.g., health literacy strengths) and red to indicate the lowest levels (e.g., health literacy challenges). Colour-coded scores are only relative to other levels in the same row (i.e., scores in Scale 1 are not comparable to those in Scale 2) and do not represent predetermined high or low values for each scale.

Clusters are ordered from #1 to #14 based on the overall highest average total health literacy to the lowest across all scales.

#### Overall sample mean: Able to understand health information but can struggle to assess its quality and lacks social support for healthcare.

The high score for Scale 9 (mean 4.01) indicates that most people reported they can understand and act upon health information. Conversely, the scores for Scales 4 (mean 2.59) and 5 (mean 2.58) suggest people lack social support and struggle to assess the quality of health information they find.

#### Cluster #1: Good access to health information and services and able to navigate the healthcare system.

This cluster contained the oldest people of all the clusters (mean age 47.9 years, range 22–92 years) and almost half were in a maximum-security prison (47.8%). Of all the clusters, people in this cluster had spent the most time, on average, in custody (mean 4.4 years). The high mean scores for Scale 3 (mean 3.79) indicate that almost all people in this cluster reported they actively manage their health, and Scale 1 (mean 3.51) suggests they felt healthcare providers understood them.

#### Cluster #2: Able to easily engage with healthcare providers and understand health information.

This cluster had the highest proportion of people who self-identified as Aboriginal (45.0%) and a high percentage of people on remand (45.0%). People in this cluster scored highest among all clusters for Scales 6 (mean 4.74), Scale 7 (mean 4.58), Scale 8 (mean 4.49), and Scale 9 (mean 4.83), indicating that most people in this cluster reported that it is always easy to engage with healthcare providers, navigate the healthcare system and find and understand health information.

#### Cluster #3: Actively managing health but can struggle to navigate the healthcare system and find good health information.

Most people in this cluster (85.2% male) had completed year 11 or 12 at high school (51.9%) and had the highest proportion of people who self-reported health conditions (70.4%); however, they reported the lowest proportion of people who stated English primarily being spoken at home (77.8%). They scored highest amongst all clusters for Scale 4—Social support for health (mean 3.13) and second highest for Scale 3—Actively managing my health (mean 3.68).

#### Cluster #4: Can actively engage with health care providers and understand health information but is not actively managing health.

This cluster was mainly comprised of males (88.9%) who were sentenced (85.2%) and had the lowest number of people who self-identified as Aboriginal (11.1%) of all clusters. People in this cluster tended not to actively manage their health (Scale 3 mean 2.42) despite reporting being able to actively engage with healthcare providers (Scale 6 mean 4.08) and understand health information (Scale 9 mean 4.36).

#### Cluster #5: Able to understand health information and actively engage with healthcare providers but does not always feel like having social support for health.

This was one of only two large clusters, which is mainly male (84.6%), with just under one-fifth of people identifying as Aboriginal (19.5%). They felt they didn’t always have social support for health (Scale 4 mean 2.85). However, they could engage with healthcare providers (Scale 6 mean 3.84) and understand health information (Scale 9 mean 4.12).

#### Cluster #6: Actively engaging with healthcare providers but can struggle to appraise and find good health information.

This was the largest cluster, mainly comprised of males (89.0%) with a mean age of 43.4 years. Over two-thirds of people (67.1%) were serving a custodial sentence and half (50.0%) were housed in a minimum-security prison. People in this cluster reported actively managing their health (Scale 3 mean 2.89) but did have difficulties appraising (Scale 5 mean 2.48) and finding health information (Scale 8 mean 3.40).

#### Cluster #7: Understands and manages health but struggles with finding and appraising health information and navigation of the healthcare system.

This cluster (25.9% female) had the highest number of people who completed year 11 or 12 at high school (55.6%), and over two-thirds (66.7%) self-reported a health condition. They felt they could actively manage their health (Scale 3 mean 3.26). Scores in Scale 5 (mean 2.22), Scale 8 (mean 2.60) and Scale 7 (mean 2.67) indicate most people in this cluster reported they struggled to find and appraise health information and to find healthcare services.

#### Cluster #8: Understood and supported by healthcare providers but can struggle to navigate the healthcare system and to find and understand health information.

The people in this cluster (30.6% female) had the lowest proportion of self-reported health conditions (38.9%) among all clusters. They mostly reported feeling understood and supported by healthcare providers (Scale 1 mean 2.97) but struggled to navigate the health system (Scale 7 mean 2.78) and find good health information (Scale 8 mean 2.73).

#### Cluster #9: Can actively manage health but has low support from healthcare provider and limited ability to navigate the healthcare system.

This is a relatively large cluster, with nearly one-tenth (9.3%; 25.0% females) of all participants. This cluster was the most educated with just under one-fifth (18.1%) of people reporting they had completed a Bachelor’s degree. They reported actively managing their health (Scale 3 mean 3.09). They did not feel supported by healthcare providers (Scale 1 mean 2.35) and struggled to navigate the healthcare system (Scale 7 mean 2.38).

#### Cluster #10: Actively managing their health and understand health information but can have difficulty finding and appraising health information.

This cluster had the lowest number of females (9.4%) among all the clusters. Over half (53.1%) self-reported having a health condition and 15.6% had completed a Bachelor’s degree. They could understand health information (Scale 9 mean 4.23) and actively manage their health (Scale 3 mean 2.91). Despite this, they reported difficulties finding (Scale 8 mean 2.36) and appraising health information (Scale 5 mean 2.03).

#### Cluster #11: Limited ability to access healthcare, lack social support and not managing health.

The people in this cluster were relatively young (37.2 years); one-third (35.7%) identified as Aboriginal and had low education levels (none had completed higher than year ten at high school). They reported not actively managing their health (Scale 3 mean 2.24), lacking social support for health (Scale 4 mean 2.47), and not being able to engage with healthcare providers (Scale 6 mean 2.50).

#### Cluster #12: Actively managing health but can struggle to navigate healthcare system and limited ability to engage with healthcare providers and to find health information.

The people in this cluster had the lowest mean age among all clusters (35.9 years; range 22–59 years), had spent the least amount of time in custody (1.3 years) and were reasonably educated (53.1% completed year 11 or 12 at high school and 15.6% completed a Bachelor’s degree). These participants reported actively managing their health (Scale 3 mean 3.01). However, they had experienced particular challenges engaging with healthcare providers (Scale 6 mean 2.05), navigating the system (Scale 7 mean 1.84), and finding health information (Scale 8 mean 1.92).

#### Cluster #13: Actively managing health but can struggle to access health care providers and has a lack of social support.

People in this cluster (female 29.2%) had spent large periods of time in custody (average of 4.3 years) and just under one-fifth (16.7%) completed a Bachelor’s degree. They reported quite low scores on 7 of the 9 scales, in particular in feeling understood and supported by (Scale 1 mean 1.41), and engaging with healthcare providers (Scale 6 mean 1.75). However, they reported two substantial strengths: they felt they could understand health information (Scale 9 mean 4.03) and actively managed their health (Scale 3 mean 3.24).

#### Cluster #14: Limited ability to find and appraise health information, struggles to navigate healthcare system, and lack social support for health.

This is the smallest cluster (1.3%), with one-third being female (33.3%), two-thirds not serving a custodial sentence (66.7%), and all spoke English primarily at home. These participants reported experiencing challenges across all HLQ scales and had the lowest scores for most scales.

### Activity 1: Cluster analyses of HLQ data – cluster solution for people who identified as Aboriginal

A 9-cluster solution was selected as the optimal cluster solution for the sample of people who identify as Aboriginal. Table 2 describes the HLQ scale scores and associated participant demographic data for the cluster solutions. Each cluster is described below.

**Table 2:** Aboriginal identity mean and health literacy profiles based on the 9-cluster solution.

Clusters		Mean	A1	A2	A3	A4	A5	A6	A7	A8	A9
% of participants in the cluster		**100**	~ 5	14.4	21.6	12.4	7.2	21.6	~ 5	8.2	6.2
**Within cluster HLQ scale mean score (SD)**											
1. Feeling understood and supported by health care providers	Score range: 1–4	**2.65** **(0.62)**	3.56 (0.43)	3.05 (0.30)	2.81 (0.33)	2.85 (0.33)	2.82 (0.31)	2.56 (0.45)	1.44 (0.52)	2.31 (0.46)	1.46 (0.66)
2. Having sufficient information to manage my health		**2.73** **(0.52)**	3.75 (0.29)	3.02 (0.25)	2.83 (0.28)	3.00 (0.15)	3.04 (0.22)	2.51 (0.43)	2.56 (0.55)	2.28 (0.28)	1.67 (0.41)
3. Actively managing my health		**2.96** **(0.45)**	3.80 (0.23)	2.94 (0.18)	2.74 (0.31)	3.28 (0.35)	2.63 (0.34)	3.07 (0.32)	3.60 (0.23)	2.58 (0.51)	2.60 (0.54)
4. Social support for health		**2.57** **(0.49)**	3.00 (0.49)	2.99 (0.24)	2.51 (0.33)	3.02 (0.28)	2.49 (0.20)	2.29 (0.28)	2.25 (1.06)	2.68 (0.37)	1.73 (0.33)
5. Appraisal of health information		**2.55** **(0.45)**	3.20 (0.16)	2.94 (0.21)	2.71 (0.29)	2.62 (0.32)	2.37 (0.24)	2.30 (0.33)	3.00 (0.28)	2.25 (0.30)	1.70 (0.24)
6. Ability to actively engage with healthcare providers	Score range: 1–5	**3.32** **(0.93)**	4.45 (0.57)	4.64 (0.28)	3.65 (0.33)	3.42 (0.39)	3.29 (0.34)	2.96 (0.54)	1.45 (0.44)	2.40 (0.43)	1.90 (0.28)
7. Navigating the healthcare system		**3.15** **(0.92)**	4.63 (0.25)	4.35 (0.34)	3.61 (0.34)	3.31 (0.44)	2.95 (0.30)	2.57 (0.52)	1.96 (0.28)	2.29 (0.49)	1.58 (0.40)
8. Ability to find good health information		**3.07** **(0.89)**	4.10 (0.60)	3.87 (0.78)	3.74 (0.40)	3.28 (0.59)	2.71 (0.40)	2.59 (0.40)	2.25 (0.53)	2.03 (0.51)	1.70 (0.49)
9. Understand health information well enough to know what to do		**3.85** **(0.77)**	4.85 (0.10)	4.70 (0.30)	4.00 (0.32)	3.70 (0.48)	2.69 (0.66)	4.01 (0.49)	3.80 (0.95)	3.05 (0.58)	2.93 (0.83)
**Within cluster sociodemographic characteristics**											
Mean age (years)		**37.2**	34.5	42.5	36.9	34.8	31.5	40.6	39.0	31.4	34.4
Age categories											
18 to 29 years (%)		**30.9**	~ 30.0	21.4	38.1	16.7	57.1	28.6	~ 30.0	37.5	33.3
30 to 45 years (%)		**44.3**	~ 80.0	21.4	38.1	75.0	28.6	42.9	~ 50.0	62.5	33.3
45 to 59 years (%)		**20.6**	0.0	50.0	19.0	8.3	14.3	19.0	~ 30.0	0.0	33.3
Older than 60 years (%)		**4.1**	0.0	7.1	4.8	0.0	0.0	9.5	0.0	0.0	0.0
Females (%)		**26.8**	~ 30.0	35.7	19.0	25.0	0.0	33.3	~ 30.0	37.5	33.3
English is primary language spoken at home (%)		**100**	100.0	100.0	100.0	100.0	100.0	100.0	100.0	100.0	100.0
Self-reported a health condition (%)		**53.6**	~ 50.0	71.4	42.9	41.7	14.3	57.1	~ 50.0	87.5	66.7
Completed year 10 or below at high school (%)		**69.1**	~ 80.0	78.6	81.0	100.0	85.7	90.5	~ 80.0	75.0	100.0
Completed a Bachelor’s degree or above (%)		**3.1**	0.0	7.1	0.0	0.0	0.0	9.5	0.0	0.0	0.0
Mean time in custody (years)		**2.0**	1.7	3.1	2.1	2.4	2.2	1.6	2.8	1.7	0.5
Serving a custodial sentence (%)		**70.1**	100.0	78.0	76.2	58.3	85.7	66.7	100.0	62.5	16.7
Security classification											
Minimum (%)		**39.2**	~ 50.0	35.7	47.6	41.7	28.6	47.6	~ 50.0	12.5	16.7
Medium (%)		**19.6**	0.0	0.0	23.8	25.0	28.6	19.0	0.0	50.0	16.7
Maximum (%)		**41.2**	~ 50.0	64.3	28.6	33.3	42.9	33.3	~ 50.0	37.5	66.7

Notes: The colour coding of cells within clusters uses green to indicate the highest levels (e.g., health literacy strengths) and red to indicate the lowest levels (e.g., health literacy challenges). Colour-coded scores are only relative to other levels in the same row (i.e., scores in Scale 1 are not comparable to those in Scale 2) and do not represent predetermined high or low values for each scale. Raw counts are not presented due to low numbers for some of the cells, with percentages for these cells presented as approximates to reduce re-identification risks.

Clusters are ordered from A1 to A9 based on the overall highest average total health literacy to the lowest across all scales.

#### Aboriginal identity mean: Actively managing health, can understand health information but can struggle to find and appraise new health information.

The score for Scale 3 (mean 2.96) indicates that a majority people who self-identified as Aboriginal feel they actively manage their health and Scale 9 (mean 3.85) suggests most can understand and apply health information. However, the scores on Scale 8 (mean 3.07) and Scale 5 (2.55) suggest they struggle to find good and appraise new health information.

#### Cluster A1: Actively managing health, able to engage with healthcare providers, and has a good understanding of health information.

This is one of the smallest clusters, with all participants serving a custodial sentence. Amongst all clusters, they scored highest on all HLQ scales except for Scale 4 (mean 3.0) and Scale 6 (mean 4.45), indicating they had health literacy strengths across all the HLQ scales when they manage their health.

#### Cluster A2: Able to engage with healthcare providers and understand health information.

On average, people in this cluster were the oldest (mean age 42.5 years) and had spent the most time in custody (3.1 years) among all clusters. They felt they could actively engage with health care providers (Scale 6 mean 4.64) and understand health information (Scale 9 mean 4.70).

#### Cluster A3: Able to understand health information but lacking social and health provider support.

This cluster was the equal largest, with Cluster A6, of all clusters (n = 21; 21.7%). It is mainly male (81.0%), has low levels of high school education (only 19.0% completed year 11 or 12 at high school), and over half did not report having a health condition (57.1%). People in this cluster felt they could understand health information (Scale 9 mean 4.00). However, they indicated they did not always feel supported by healthcare providers (Scale 1 mean 2.81) and lacked social support for health (Scale 4 mean 2.51).

#### Cluster A4: Actively manage health but struggle to find good health information and navigate the healthcare system.

The people in this cluster (75.0% males) were relatively young (mean age of 34.3 years) and had not completed year 10 at high school or had any post-high school qualifications. Participants felt they could actively manage their health (Scale 3 mean score of 3.28). However, they reported some struggles with navigating the healthcare system (Scale 7 mean 3.31) and finding good health information (Scale 8 mean 3.28).

#### Cluster A5: Struggle to engage with the healthcare system and appraise and understand health information.

This was a young (average age 31.5 years) all-male cluster, with most participants currently serving a custodial sentence (85.7%). Among all clusters, people in this cluster scored lowest on Scale 9 (mean 2.69). People in this cluster reported they struggled to find (Scale 8 mean 2.71) and appraise (Scale 5 mean 2.37) health information. Despite this, they felt they had sufficient information to manage their health (Scale 2 mean 3.04).

#### Cluster A6: Actively manage health but lack social support and struggle to navigate the healthcare system.

This cluster was the equal largest with Cluster A3 (n = 21; 21.7%). One-third were female (33.3%), and just under one-tenth had completed a Bachelor’s degree (9.5%). People in this cluster reported actively managing their health (Scale 3 mean 3.07); however, they felt they lacked social support for health (Scale 4 mean 2.29) and struggled to navigate the healthcare system (Scale 7 mean 2.57).

#### Cluster A7: Can understand health information well but do not have support from healthcare providers and struggle to engage with healthcare providers.

This is the smallest cluster (tied with Cluster A1). All people in this cluster were serving a custodial sentence, and none had completed post-high school qualifications. Among all clusters, people in this cluster scored lowest on Scale 1 (mean 1.44) and Scale 6 (mean 1.45). Despite this, they scored highly on Scale 3 (mean 3.60) and Scale 5 (mean 3.00) compared to all other clusters.

#### Cluster A8: Lack sufficient health information and have difficulty to find good health information.

Among all clusters, people in this cluster had the youngest mean age (31.4 years), the highest proportion of females (37.5%), and of people who self-reported having a health condition (87.5%). They felt they did not have sufficient information to manage their health (Scale 2 mean 2.28) and struggled to find good health information (Scale 8 mean 2.03).

#### Cluster A9: Limited ability to navigate the system and lack social and healthcare support.

People in this cluster (males 66.7%) were mainly on remand (83.3%) and had, on average, spent the least amount of time in custody (0.5 years). Like most clusters, people had higher scores for Scale 3 (mean 2.60) and Scale 9 (mean 2.93); however, they reported health literacy challenges across all HLQ scales.

### Activity 2: Semi-structured interviews

Ten respondents, seven males and three females aged from 23 to 84 years, participated in semi-structured interviews and provided further insights into their experiences of using health services in NSW prisons.

### Activity 3: Vignettes developed

**Fig 2:**
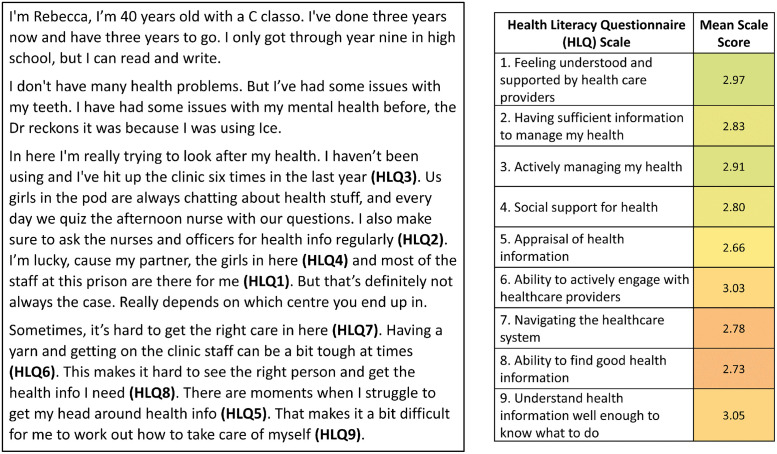
An example of a vignette representing Cluster #8 and the associated HLQ scale scores. Note: HLQ =  Health Literacy Questionnaire Scale. HLQ Scales 1–5 are rated on a 4-point agreement scale (1: strongly disagree to 4: strongly agree) and Scales 6–9 are rated on a 5-point ease scale (1: cannot do or always difficult to 5: always easy). HLQ scale numbers (e.g., HLQ 1) were not included in the vignettes presented in the ideas generation workshops and yarning circles. The scale numbers and accompanying scale mean scores are shown here to assist in examining where the vignette text comes from. The colour coding of cells for mean scale scores uses green to indicate the highest levels (e.g., health literacy strengths) and red to indicate the lowest levels (e.g., health literacy challenges), which are relative to the scale scores across clusters.

**Fig 3:**
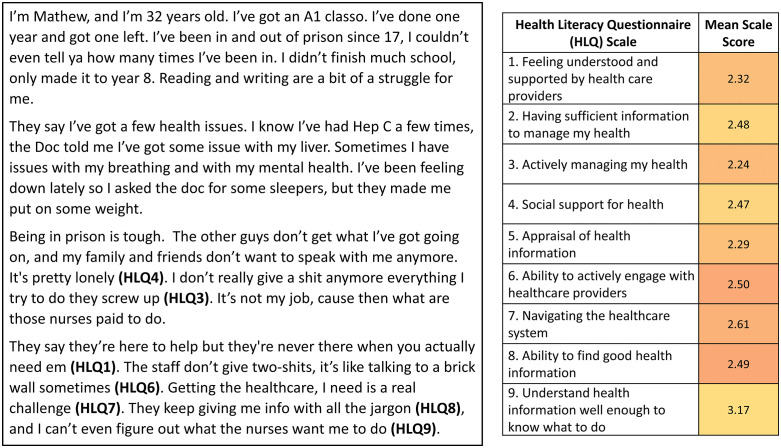
An example of a vignette representing Cluster #11 and the associated HLQ scale scores. Note: HLQ =  Health Literacy Questionnaire Scale. HLQ Scales 1–5 are rated on a 4-point agreement scale (1: strongly disagree to 4: strongly agree) and Scales 6–9 are rated on a 5-point ease scale (1: cannot do or always difficult to 5: always easy). HLQ scale numbers (e.g., HLQ 1) were not included in the vignettes presented in the ideas generation workshops and yarning circles. The scale numbers and accompanying scale mean scores are shown here to assist in examining where the vignette text comes from. The colour coding of cells for mean scale scores uses green to indicate the highest levels (e.g., health literacy strengths) and red to indicate the lowest levels (e.g., health literacy challenges), which are relative to the scale scores across clusters.

**Fig 4:**
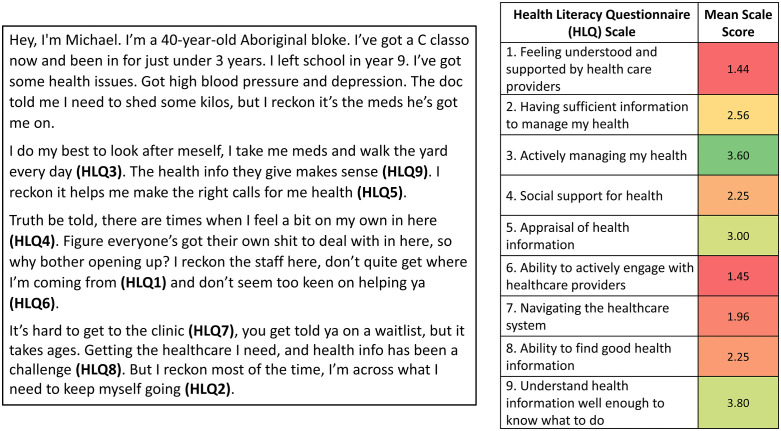
An example of a vignette representing Aboriginal identity Cluster A7 and the associated HLQ scale scores. Note: HLQ =  Health Literacy Questionnaire Scale. HLQ Scales 1–5 are rated on a 4-point agreement scale (1: strongly disagree to 4: strongly agree) and Scales 6–9 are rated on a 5-point ease scale (1: cannot do or always difficult to 5: always easy). HLQ scale numbers (e.g., HLQ 1) were not included in the vignettes presented in the ideas generation workshops and yarning circles. The scale numbers and accompanying scale mean scores are shown here to assist in examining where the vignette text comes from. The colour coding of cells for mean scale scores uses green to indicate the highest levels (e.g., health literacy strengths) and red to indicate the lowest levels (e.g., health literacy challenges), which are relative to the scale scores across clusters.

**Fig 5:**
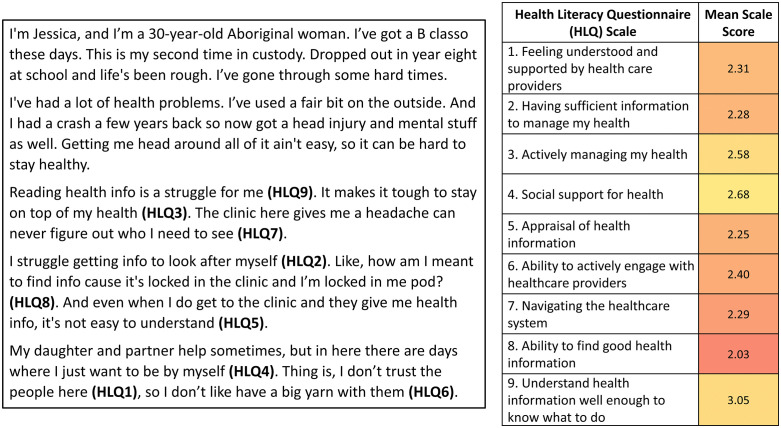
An example of a vignette representing Aboriginal Identity Cluster A8 and the associated HLQ scale scores. Note: HLQ =  Health Literacy Questionnaire Scale. HLQ Scales 1–5 are rated on a 4-point agreement scale (1: strongly disagree to 4: strongly agree) and Scales 6–9 are rated on a 5-point ease scale (1: cannot do or always difficult to 5: always easy). HLQ scale numbers (e.g., HLQ 1) were not included in the vignettes presented in the ideas generation workshops and yarning circles. The scale numbers and accompanying scale mean scores are shown here to assist in examining where the vignette text comes from. The colour coding of cells for mean scale scores uses green to indicate the highest levels (e.g., health literacy strengths) and red to indicate the lowest levels (e.g., health literacy challenges), which are relative to the scale scores across clusters.

Based on the cluster analyses and ten semi-structured interviews, 23 vignettes were developed to represent a typical person from each cluster. Throughout the co-design and stakeholder engagement (n = 34), stakeholders advocated for vignettes to be rewritten and trialled in first person to increase the likelihood they would resonate with participants in the next stages of the project. Therefore, in preparation for vignettes being presented to various stakeholder groups, both first and third-person vignettes were developed. See Fig 2 for an example of Cluster #8 (*Understood and supported by healthcare providers but can struggle to navigate the healthcare system and to find and understand health information*), Fig 3 for an example of Cluster #11 (*Limited ability to access healthcare, lack social support and not managing health*), Fig 4 for an example of Aboriginal Identity Cluster A7 (*Can understand health information well but do not have support from healthcare providers and struggle to engage with healthcare providers*), Fig 5 for an example of Aboriginal Identity Cluster A8 (*Lack sufficient health information and have difficulty to find good health information*) and S1-S19 Figs for all other clusters.

## Discussion

This study is the first to apply cluster analysis to multidimensional health literacy data from people in prison and to translate the identified health literacy profiles into co-designed, evidence-based vignettes. Rather than treating people in prison as a homogenous group or relying on average scores, our findings demonstrate substantial heterogeneity in health literacy strengths and challenges experienced by people in NSW prisons. The diversity exhibited in the health literacy profiles and subsequent vignettes provides a deeper understanding of the challenges people may face when accessing, understanding and using health information and services in NSW prisons, with critical implications for intervention design. The findings of this study are novel and can be used to inform the development of localised evidence-based health literacy actions and solutions to improve how people engage with prison health services.

### How cluster analysis can move us from means to meaningful

The use of cluster analysis is growing in popularity within the healthcare and intervention development fields [21]. Its usefulness in providing more meaningful and person-centred information has been demonstrated in cancer research [39], post-surgery outcomes [40,41] and health literacy development projects [21,22]. Cluster analysis in this study has unmasked variations in the health literacy strengths and challenges of people in prison. Cluster analysis for the overall sample revealed that over two-thirds (68.8%) of participants were experiencing some form of challenge on at least one dimension of health literacy. Similarly, the Aboriginal identity analysis revealed that 50% were experiencing one or more health literacy dimension challenges. These findings highlight that mean-based approaches can misrepresent population realities, as examining the mean HLQ scale scores alone would suggest that everyone in both populations of interest, to some degree, experiences similar health literacy challenges. By contrast, the cluster-based approach reveals substantial heterogeneity in health literacy profiles and has uncovered previously unobserved variations.

The analyses also highlighted that different health literacy strengths and challenges can be found among clusters. For example, Cluster #4 (*Can actively engage with health care providers and understand health information but is not actively managing health*) indicated that people had many strengths across the dimensions of health literacy; however, they still experienced challenges, as evidenced by the lower mean score on Scale 3. Conversely, the analyses also revealed clusters of people who still have strengths despite experiencing several health literacy challenges. For example, Clusters A6 (*Actively manage health but lack social support and struggle to navigate the healthcare system*) and A7 (*Can understand health information well but do not have support from healthcare providers and struggle to engage with healthcare providers*) have strengths, particularly around actively managing their health (Scale 3) and understanding health information (Scale 9) despite reported challenges across other dimensions. Thus, cluster analysis demonstrates that the identified groups within the NSW prison population are simultaneously experiencing health literacy strengths and challenges. These findings show that mean-based approaches can mask these potential mechanisms of health literacy which can be leveraged to improve how people in prison access and use health information and services.

### Health literacy proxies do not provide an accurate picture of population diversity

Health literacy scores have long been associated with poorer health outcomes [42], education level and lower socio-economic position [43,44]. These associations are usually drawn from a specific sample following an inferential and descriptive analysis. However, a key limitation of these analyses is that they only examine whether any mean group differences exist within the sample [45]. This provides a two-dimensional view of a proxy’s impact on an individual’s or community’s health literacy. For example, people in Cluster #12 (*Actively managing health but can struggle to navigate healthcare system and limited ability to engage with healthcare providers and to find health information*) were reasonably educated and reported very low HLQ scale scores except on Scales 3 and 9. These findings show that using proxies such as education levels, which have long been associated with lower health literacy [43], may not provide an accurate picture of the strengths and challenges experienced in populations.

Similar findings were observed when examining the participant demographic sub-groups. For instance, our initial descriptive study, which relied on comparisons of scale means between demographic groups, found that females had several health literacy challenges compared with males [20]. In contrast, the current cluster-based analysis showed that some clusters with higher proportions of females were not always among those reporting several health literacy challenges. For example, clusters A2 (*Able to engage with healthcare providers and understand health information*), #7 (*Understands and manages health but struggles with finding and appraising health information and navigation of the healthcare system*) and #8 (*Understood and supported by healthcare providers but can struggle to navigate the healthcare system and to find and understand health information*), which have higher proportions of females, had several strengths across health literacy dimensions. Highlighting that if we treat sub-groups as homogenous and only use the mean scores, we could mask any variation or the heterogeneity of health literacy patterns within these groups [21]. Which, in turn, will inevitably prevent the specific needs of people within these sub-groups, such as some females, from being met. We therefore caution researchers against relying solely on proxies in health literacy research and recommend the use of alternative analyses, such as cluster analysis, that provide more profound and meaningful insights into the experiences of different people.

### Evidenced-based vignettes can portray the voices of those with lived experience

Consistent with the Ophelia process [23–25], cluster analysis in this study informed the development of evidence-based vignettes as a translational step rather than just an analytic one. The developed vignettes synthesise quantitative findings and qualitative interview data to portray a typical person from each identified health literacy profile, thereby supporting the practical use of findings for stakeholder engagement and intervention co-design. In this way, cluster analysis enables a more person-centred approach to exploring the health literacy strengths and challenges of differing groups of people. Across two analyses, our study revealed diverse health literacy profiles of people in NSW prisons. The results from these analyses then formed the basis for representing the voice and sharing the stories of people within each cluster. The addition of real-life experiences from semi-structured interviews and the iterative development process not only strengthened this voice but ensured we were true to the experience of people with lived experience. Further, it enables the voices of people in prison to be heard and allows researchers to present findings in a lively way. By translating mean HLQ scores through cluster analysis into meaningful stories that individuals can relate to, the vignettes have the potential to create engaging discussions. Moreover, developing vignettes depicting people from specific sub-groups, for example, sex-specific or Aboriginal-specific vignettes, may create meaningful discussions relevant to the needs of these groups of people.

### Strengths and limitations

A strength of this study is using a more robust analysis (i.e., cluster analysis) combined with semi-structured interview data to transform HLQ data into meaningful vignettes. Further, the iterative approach throughout vignette development ensured that various stakeholders were involved in co-designing and improving these vignettes. For example, the advocacy by stakeholders to re-write these vignettes in first person to improve the resonation and engagement in future stages of the project.

The study does have some limitations that need acknowledgement. The voices of people from CALD backgrounds and people in rural correctional centres may not be represented in the participant sample. Moreover, health condition data was not included in the dataset provided due to limitations noted in the original needs assessment [20], which may limit the generalisability of our study findings. To minimise this limitation, health condition data and information regarding CALD populations were drawn on through the semi-structured interviews. Further, the iterative vignette development process ensured the vignettes depicted a range of people with varying backgrounds and health needs.

### Implications of the study

The vignettes developed in this study will now be applied in a series of ideas generation workshops and yarning circles (i.e., a form of Aboriginal peoples’ conversational data collection) with various stakeholders, such as people in prison and staff, to harness their local wisdom to develop actions and solutions for health literacy-informed interventions. These co-design activities will systematically enable the voice of these stakeholders to generate fit-for-purpose localised actions and solutions through engagement with the stories presented in the vignettes to understand and improve the delivery of information, services, and resources for people in prison.

## Conclusion

This novel study applied cluster analysis to HLQ data from people in prison to translate mean scale data into co-designed, evidence-based vignettes. Exploring the heterogeneity of health literacy strengths and challenges in the NSW prison population has demonstrated substantial variation that is masked by mean-based approaches. Cluster analysis enabled us to move from just reporting means to gaining meaningful insights into the health literacy profiles of people in prison. Our findings are important as they highlight that using the mean or proxies does not provide a picture of population diversity, which may inadvertently exclude or disadvantage population sub-groups. Consistent with the Ophelia process, the vignettes are expected to stimulate lively discussion to generate localised, fit-for-purpose health literacy-informed interventions to address health inequities in NSW prisons.

## Supporting information

S1 File Example Interview Schedule for Vignette Development. (PDF)

S1 Fig Example of vignette representing Cluster #1. Note: HLQ =  Health Literacy Questionnaire Scale. HLQ Scales 1–5 are rated on a 4-point agreement scale (1: strongly disagree to 4: strongly agree) and Scales 6–9 are rated on a 5-point ease scale (1: cannot do or always difficult to 5: always easy). HLQ scale numbers (e.g., HLQ 1) were not included in the vignettes presented in the ideas generation workshops and yarning circles. The scale numbers and accompanying scale mean scores are shown here to assist in examining where the vignette text comes from. The colour coding of cells for mean scale scores uses green to indicate the highest levels (e.g., health literacy strengths) and red to indicate the lowest levels (e.g., health literacy challenges), which are relative to the scale scores across clusters.
(TIF)

S2 Fig Example of vignette representing Cluster #2. Note: HLQ =  Health Literacy Questionnaire Scale. HLQ Scales 1–5 are rated on a 4-point agreement scale (1: strongly disagree to 4: strongly agree) and Scales 6–9 are rated on a 5-point ease scale (1: cannot do or always difficult to 5: always easy). HLQ scale numbers (e.g., HLQ 1) were not included in the vignettes presented in the ideas generation workshops and yarning circles. The scale numbers and accompanying scale mean scores are shown here to assist in examining where the vignette text comes from. The colour coding of cells for mean scale scores uses green to indicate the highest levels (e.g., health literacy strengths) and red to indicate the lowest levels (e.g., health literacy challenges), which are relative to the scale scores across clusters.
(TIF)

S3 Fig Example of vignette representing Cluster #3. Note: HLQ =  Health Literacy Questionnaire Scale. HLQ Scales 1–5 are rated on a 4-point agreement scale (1: strongly disagree to 4: strongly agree) and Scales 6–9 are rated on a 5-point ease scale (1: cannot do or always difficult to 5: always easy). HLQ scale numbers (e.g., HLQ 1) were not included in the vignettes presented in the ideas generation workshops and yarning circles. The scale numbers and accompanying scale mean scores are shown here to assist in examining where the vignette text comes from. The colour coding of cells for mean scale scores uses green to indicate the highest levels (e.g., health literacy strengths) and red to indicate the lowest levels (e.g., health literacy challenges), which are relative to the scale scores across clusters.
(TIF)

S4 Fig Example of vignette representing Cluster #4. Note: HLQ =  Health Literacy Questionnaire Scale. HLQ Scales 1–5 are rated on a 4-point agreement scale (1: strongly disagree to 4: strongly agree) and Scales 6–9 are rated on a 5-point ease scale (1: cannot do or always difficult to 5: always easy). HLQ scale numbers (e.g., HLQ 1) were not included in the vignettes presented in the ideas generation workshops and yarning circles. The scale numbers and accompanying scale mean scores are shown here to assist in examining where the vignette text comes from. The colour coding of cells for mean scale scores uses green to indicate the highest levels (e.g., health literacy strengths) and red to indicate the lowest levels (e.g., health literacy challenges), which are relative to the scale scores across clusters.
(TIF)

S5 Fig Example of vignette representing Cluster #5. Note: HLQ =  Health Literacy Questionnaire Scale. HLQ Scales 1–5 are rated on a 4-point agreement scale (1: strongly disagree to 4: strongly agree) and Scales 6–9 are rated on a 5-point ease scale (1: cannot do or always difficult to 5: always easy). HLQ scale numbers (e.g., HLQ 1) were not included in the vignettes presented in the ideas generation workshops and yarning circles. The scale numbers and accompanying scale mean scores are shown here to assist in examining where the vignette text comes from. The colour coding of cells for mean scale scores uses green to indicate the highest levels (e.g., health literacy strengths) and red to indicate the lowest levels (e.g., health literacy challenges), which are relative to the scale scores across clusters.
(TIF)

S6 Fig Example of vignette representing Cluster #6. Note: HLQ =  Health Literacy Questionnaire Scale. HLQ Scales 1–5 are rated on a 4-point agreement scale (1: strongly disagree to 4: strongly agree) and Scales 6–9 are rated on a 5-point ease scale (1: cannot do or always difficult to 5: always easy). HLQ scale numbers (e.g., HLQ 1) were not included in the vignettes presented in the ideas generation workshops and yarning circles. The scale numbers and accompanying scale mean scores are shown here to assist in examining where the vignette text comes from. The colour coding of cells for mean scale scores uses green to indicate the highest levels (e.g., health literacy strengths) and red to indicate the lowest levels (e.g., health literacy challenges), which are relative to the scale scores across clusters.
(TIF)

S7 Fig Example of vignette representing Cluster #7. Note: HLQ =  Health Literacy Questionnaire Scale. HLQ Scales 1–5 are rated on a 4-point agreement scale (1: strongly disagree to 4: strongly agree) and Scales 6–9 are rated on a 5-point ease scale (1: cannot do or always difficult to 5: always easy). HLQ scale numbers (e.g., HLQ 1) were not included in the vignettes presented in the ideas generation workshops and yarning circles. The scale numbers and accompanying scale mean scores are shown here to assist in examining where the vignette text comes from. The colour coding of cells for mean scale scores uses green to indicate the highest levels (e.g., health literacy strengths) and red to indicate the lowest levels (e.g., health literacy challenges), which are relative to the scale scores across clusters.
(TIF)

S8 Fig Example of vignette representing Cluster #9. Note: HLQ =  Health Literacy Questionnaire Scale. HLQ Scales 1–5 are rated on a 4-point agreement scale (1: strongly disagree to 4: strongly agree) and Scales 6–9 are rated on a 5-point ease scale (1: cannot do or always difficult to 5: always easy). HLQ scale numbers (e.g., HLQ 1) were not included in the vignettes presented in the ideas generation workshops and yarning circles. The scale numbers and accompanying scale mean scores are shown here to assist in examining where the vignette text comes from. The colour coding of cells for mean scale scores uses green to indicate the highest levels (e.g., health literacy strengths) and red to indicate the lowest levels (e.g., health literacy challenges), which are relative to the scale scores across clusters.
(TIF)

S9 Fig Example of vignette representing Cluster #10. Note: HLQ =  Health Literacy Questionnaire Scale. HLQ Scales 1–5 are rated on a 4-point agreement scale (1: strongly disagree to 4: strongly agree) and Scales 6–9 are rated on a 5-point ease scale (1: cannot do or always difficult to 5: always easy). HLQ scale numbers (e.g., HLQ 1) were not included in the vignettes presented in the ideas generation workshops and yarning circles. The scale numbers and accompanying scale mean scores are shown here to assist in examining where the vignette text comes from. The colour coding of cells for mean scale scores uses green to indicate the highest levels (e.g., health literacy strengths) and red to indicate the lowest levels (e.g., health literacy challenges), which are relative to the scale scores across clusters.
(TIF)

S10 Fig Example of vignette representing Cluster #12. Note: HLQ =  Health Literacy Questionnaire Scale. HLQ Scales 1–5 are rated on a 4-point agreement scale (1: strongly disagree to 4: strongly agree) and Scales 6–9 are rated on a 5-point ease scale (1: cannot do or always difficult to 5: always easy). HLQ scale numbers (e.g., HLQ 1) were not included in the vignettes presented in the ideas generation workshops and yarning circles. The scale numbers and accompanying scale mean scores are shown here to assist in examining where the vignette text comes from. The colour coding of cells for mean scale scores uses green to indicate the highest levels (e.g., health literacy strengths) and red to indicate the lowest levels (e.g., health literacy challenges), which are relative to the scale scores across clusters.
(TIF)

S11 Fig Example of vignette representing Cluster #13. Note: HLQ =  Health Literacy Questionnaire Scale. HLQ Scales 1–5 are rated on a 4-point agreement scale (1: strongly disagree to 4: strongly agree) and Scales 6–9 are rated on a 5-point ease scale (1: cannot do or always difficult to 5: always easy). HLQ scale numbers (e.g., HLQ 1) were not included in the vignettes presented in the ideas generation workshops and yarning circles. The scale numbers and accompanying scale mean scores are shown here to assist in examining where the vignette text comes from. The colour coding of cells for mean scale scores uses green to indicate the highest levels (e.g., health literacy strengths) and red to indicate the lowest levels (e.g., health literacy challenges), which are relative to the scale scores across clusters.
(TIF)

S12 Fig Example of vignette representing Cluster #14. Note: HLQ =  Health Literacy Questionnaire Scale. HLQ Scales 1–5 are rated on a 4-point agreement scale (1: strongly disagree to 4: strongly agree) and Scales 6–9 are rated on a 5-point ease scale (1: cannot do or always difficult to 5: always easy). HLQ scale numbers (e.g., HLQ 1) were not included in the vignettes presented in the ideas generation workshops and yarning circles. The scale numbers and accompanying scale mean scores are shown here to assist in examining where the vignette text comes from. The colour coding of cells for mean scale scores uses green to indicate the highest levels (e.g., health literacy strengths) and red to indicate the lowest levels (e.g., health literacy challenges), which are relative to the scale scores across clusters.
(TIF)

S13 Fig Example of vignette representing Cluster A1. Note: HLQ =  Health Literacy Questionnaire Scale. HLQ Scales 1–5 are rated on a 4-point agreement scale (1: strongly disagree to 4: strongly agree) and Scales 6–9 are rated on a 5-point ease scale (1: cannot do or always difficult to 5: always easy). HLQ scale numbers (e.g., HLQ 1) were not included in the vignettes presented in the ideas generation workshops and yarning circles. The scale numbers and accompanying scale mean scores are shown here to assist in examining where the vignette text comes from. The colour coding of cells for mean scale scores uses green to indicate the highest levels (e.g., health literacy strengths) and red to indicate the lowest levels (e.g., health literacy challenges), which are relative to the scale scores across clusters.
(TIF)

S14 Fig Example of vignette representing Cluster A2. Note: HLQ =  Health Literacy Questionnaire Scale. HLQ Scales 1–5 are rated on a 4-point agreement scale (1: strongly disagree to 4: strongly agree) and Scales 6–9 are rated on a 5-point ease scale (1: cannot do or always difficult to 5: always easy). HLQ scale numbers (e.g., HLQ 1) were not included in the vignettes presented in the ideas generation workshops and yarning circles. The scale numbers and accompanying scale mean scores are shown here to assist in examining where the vignette text comes from. The colour coding of cells for mean scale scores uses green to indicate the highest levels (e.g., health literacy strengths) and red to indicate the lowest levels (e.g., health literacy challenges), which are relative to the scale scores across clusters.
(TIF)

S15 Fig Example of vignette representing Cluster A3. Note: HLQ =  Health Literacy Questionnaire Scale. HLQ Scales 1–5 are rated on a 4-point agreement scale (1: strongly disagree to 4: strongly agree) and Scales 6–9 are rated on a 5-point ease scale (1: cannot do or always difficult to 5: always easy). HLQ scale numbers (e.g., HLQ 1) were not included in the vignettes presented in the ideas generation workshops and yarning circles. The scale numbers and accompanying scale mean scores are shown here to assist in examining where the vignette text comes from. The colour coding of cells for mean scale scores uses green to indicate the highest levels (e.g., health literacy strengths) and red to indicate the lowest levels (e.g., health literacy challenges), which are relative to the scale scores across clusters.
(TIF)

S16 Fig Example of vignette representing Cluster A4. Note: HLQ =  Health Literacy Questionnaire Scale. HLQ Scales 1–5 are rated on a 4-point agreement scale (1: strongly disagree to 4: strongly agree) and Scales 6–9 are rated on a 5-point ease scale (1: cannot do or always difficult to 5: always easy). HLQ scale numbers (e.g., HLQ 1) were not included in the vignettes presented in the ideas generation workshops and yarning circles. The scale numbers and accompanying scale mean scores are shown here to assist in examining where the vignette text comes from. The colour coding of cells for mean scale scores uses green to indicate the highest levels (e.g., health literacy strengths) and red to indicate the lowest levels (e.g., health literacy challenges), which are relative to the scale scores across clusters.
(TIF)

S17 Fig Example of vignette representing Cluster A5. Note: HLQ =  Health Literacy Questionnaire Scale. HLQ Scales 1–5 are rated on a 4-point agreement scale (1: strongly disagree to 4: strongly agree) and Scales 6–9 are rated on a 5-point ease scale (1: cannot do or always difficult to 5: always easy). HLQ scale numbers (e.g., HLQ 1) were not included in the vignettes presented in the ideas generation workshops and yarning circles. The scale numbers and accompanying scale mean scores are shown here to assist in examining where the vignette text comes from. The colour coding of cells for mean scale scores uses green to indicate the highest levels (e.g., health literacy strengths) and red to indicate the lowest levels (e.g., health literacy challenges), which are relative to the scale scores across clusters.
(TIF)

S18 Fig Example of vignette representing Cluster A6. Note: HLQ =  Health Literacy Questionnaire Scale. HLQ Scales 1–5 are rated on a 4-point agreement scale (1: strongly disagree to 4: strongly agree) and Scales 6–9 are rated on a 5-point ease scale (1: cannot do or always difficult to 5: always easy). HLQ scale numbers (e.g., HLQ 1) were not included in the vignettes presented in the ideas generation workshops and yarning circles. The scale numbers and accompanying scale mean scores are shown here to assist in examining where the vignette text comes from. The colour coding of cells for mean scale scores uses green to indicate the highest levels (e.g., health literacy strengths) and red to indicate the lowest levels (e.g., health literacy challenges), which are relative to the scale scores across clusters.
(TIF)

S19 Fig Example of vignette representing Cluster A9. Note: HLQ =  Health Literacy Questionnaire Scale. HLQ Scales 1–5 are rated on a 4-point agreement scale (1: strongly disagree to 4: strongly agree) and Scales 6–9 are rated on a 5-point ease scale (1: cannot do or always difficult to 5: always easy). HLQ scale numbers (e.g., HLQ 1) were not included in the vignettes presented in the ideas generation workshops and yarning circles. The scale numbers and accompanying scale mean scores are shown here to assist in examining where the vignette text comes from. The colour coding of cells for mean scale scores uses green to indicate the highest levels (e.g., health literacy strengths) and red to indicate the lowest levels (e.g., health literacy challenges), which are relative to the scale scores across clusters.
(TIF)
