# Supplementary material for: From means to meaningful: undertaking cluster analysis to develop health literacy profiles of people in Australian prisons
Source: PLoS One. 2026 Jun 11;21(6):e0351554. doi: 10.1371/journal.pone.0351554 (PMC13258149; doi:10.1371/journal.pone.0351554)
Supplement: S1 File — (PDF) [file pone.0351554.s001.pdf]

Example Interview Schedule for Vignette Development

Part 1 – Introduction

Hello XXX, my name is XXX and I am a researcher from [XXXX]. Can you state your name? [Confirm Identity by verbal confirmation]

Before we start the interview, I would like to remind you again that your decision to take part to not will not affect your relationship with [XXXX]. While you have agreed to take part now, you still have the right to withdraw from the interview at any time, without giving any reason. You can also withdraw within two weeks after this interview is completed. Contact details are in the Participant Information Sheet and Consent Form you received earlier.

The information you give will contribute to improving ways of people accessing and using health information and services whilst in prison.

The interview will take about 30 minutes. The questions are about the responses you gave on the questionnaire and your experiences with how you access and utilise health information, and the support you receive when accessing health care in prison. There are no right or wrong answers, so please answer the questions according to your own experience. If you need to stop at any time, just let me know.

With your permission, I will record the interview to make sure I capture your words accurately.
Before we begin, do you still have any questions about the information and consent form? Do you have any other questions about this interview?
Let me know at any time if you do not want to answer or feel uncomfortable and would like to stop the interview.
Before we being, do you still have any more questions?
(If yes, answer the questions)

(If no) Would you like to proceed? I will now start the recording. (Start Recording)

Part 2 – Interview

Ok, I am going to ask you some questions about how your experiences with how you access and utilise health information and the support you receive when accessing health care in prison. Remember, there are not right or wrong answers, so please answer the questions according to your own experience.

**Access & Use**		
**Questions**		**Response**
1	Do you access health services in prison?<br>If yes, how do you access the health services?<br>If not, why do you not access health services?	
2	Do you access health information (i.e. pamphlets, fact sheets) when in prison?<br>If yes, how do you access the health information?<br>If not, is there anything stopping you from accessing health information?	
3	How often do you access the health services in prison?<br>Would you like to have more access to the health services?<br>How can we do to help your access?	
4	When you accessing the health care, who do you normally see?<br>• Nurse<br>• Doctor<br>• Dentist<br>• Physiotherapist etc.	
**Barriers to health care**		
1	Do you have any barriers to accessing the health care you need in prison?<br>If yes, what are these barriers?<br>If not, is there anything that could be done to improve your access?	
2	You mentioned [XXXXX] as a barrier, can you tell me more about that?	
**Connecting to healthcare providers**		
1	How do you speak with healthcare providers (i.e. nurses, doctors)?<br>Do you have any suggestions on how this could be easier?	
2	When speaking with a healthcare provider, do you have any problems understanding them?<br>If yes, what are the issues you are having?<br>If not, what allows you to understand them?	
**Privacy**		
1	Do you have any concerns over the privacy when accessing healthcare in prison?	
2	Are you confident that your health information is in safe hands?	
**Support**		

1	Do you have any family or friends who can help you when accessing healthcare when you need to?	
2	How often do you have access to the people who can help you?	
**Further questions may be asked in relation to the barriers (low scores) they have as identified in their questionnaire.<br>"From your Questionnaire results, you seem to have difficulty with XX – can you explain what you mean by this?"**		
**Scale 1: Feeling understood and supported by healthcare providers**		
**Scale 2: Having sufficient information to manage my health**		
**Scale 3: Actively managing my health**		
**Scale 4: Social support for health**		
**Scale 5: Appraisal of health information**		
**Scale 6: Ability to actively engage with healthcare providers**		
**Scale 7: Navigating the healthcare system**		
**Scale 8: Ability to find good health information**		
**Scale 9: Understand health information well enough to know what to do**		

Part 3 – Conclusion

That's all there anything else that you would like to bring up?

Thank you very much for taking time to help us. I will now turn off the recording. (Stop Recording).

Interviewer to ask if the patient would like to participate in future research activities in relation to the study? (Yes or No)

Interviewer to offer a debrief with the participant.

Debriefing

**Summary of the interview**

Participant UIN: | | | | | |

Further Questions

Examples of interview questions to be supplemented, depending on participants' HLQ scores. The questions asked of participants will be guided by higher or lower Health Literacy Questionnaire scores.

**HLQ Scale**	**Higher HLQ Score**	**Lower HLQ Score**
**1. Feeling understood and supported by healthcare providers**	Do you feel understood and supported by your healthcare providers? Why do you feel this way?	Do you feel understood and supported by healthcare providers? Has it always been this way? What would help to improve these relationships?
**2. Having sufficient information to manage my health**	What helps you to feel you have enough information to manage your health and make decisions?	What would help you to feel confident that you have enough information about your health?
**3. Actively managing my health**	How did you learn to manage your health? What do you do to manage your health?	What do you do to manage your health? What do you need to help you manage your health differently?
**4. Social support for health**	What kind of help do you get from your family, friends or community for your health?	What kind of help do you get from your family, friends or community for your health?
**5. Appraisal of health information**	What helps you to understand health information? How do you work out what is best for you?	What would help you to understand health information and work out what is best for you?
**6. Ability to actively engage with healthcare providers**	How comfortable do you feel about talking about your health with healthcare providers? What has helped you with this?	How comfortable do you feel about talking with healthcare providers? What would help you to feel more comfortable to talk with them about your health?
**7. Navigating the healthcare system**	How do you find out about the health and support services that you need?	What would help you to find out about health and support services that you need?
**8. Ability to find good health information**	What has helped you to find information about your health?	What would help you to find information about your health?
**9. Understand health information well enough to know what to do**	What has helped you to understand written health information?	What would help you to understand written information about your health?
